# Association between regular exercise and asthma control among adults: The population-based Northern Finnish Asthma Study

**DOI:** 10.1371/journal.pone.0227983

**Published:** 2020-01-23

**Authors:** Maritta S. Jaakkola, Sirpa A. M. Aalto, Henna Hyrkäs-Palmu, Jouni J. K. Jaakkola

**Affiliations:** 1 Center for Environmental and Respiratory Health Research (CERH), University of Oulu, Oulu, Finland; 2 Medical Research Center Oulu (MRC Oulu), University of Oulu, Oulu, Finland; 3 Biocenter Oulu, University of Oulu, Oulu, Finland; Gazi University, Faculty of Health Sciences, TURKEY

## Abstract

Previously those with asthma were often advised to avoid strenuous exercise because of fear for exercise-induced asthmatic reactions, but recent findings suggest many beneficial effects on health related to exercise. We elaborated on the relation between regular exercise and asthma control among adults. This was a population-based cross-sectional Northern Finnish Asthma Study (NoFAS), in which altogether 1922 adult subjects 17–73 years old living in Northern Finland answered the NoFAS questionnaire. The determinant of interest was the total amount of regular exercise during leisure time, measured in hours per week and categorized into no, low (>0≤2h per week), medium (>2 ≤5h), high (>5≤10h) and very high (>10h) exercise categories. The outcome of interest was asthma control, which was assessed based on the Asthma Control Test (ACT). As statistical methods we applied analysis of variance (ANOVA) and Poisson regression. ACT score increased gradually, i.e. asthma control improved, with an increasing amount of exercise from no exercise (mean ACT = 19.4; difference from the reference: -1.57, 95% CI -2.12 to -1.01) to high exercise reference category (mean = 21.0), but was slightly lower (mean = 20.3; -0.64, 95% CI -1.27 to -0.02) in the very high exercise category. Such non-linear relation was present both in women and in men. In conclusion, we provide evidence that moderate to high regular exercise improves asthma control among adults with asthma. Advice about regular exercise should be included as an important part of asthma management for adults.

## Introduction

Bronchial asthma is a common chronic disease among adult populations in many parts of the world, especially in high and medium income countries. In the early 2010s, the global prevalence of asthma in adults was 4.3%, and in Europe it was 5.1% [1]. The prevalence of asthma among the working-age population in USA has been 7.7% [2] and that among Finnish adults 7–9% [3, 4].

In recent years there has been increasing interest in assessing potential beneficial effects that exercise may have on asthma [5]. Three recent epidemiological studies have assessed associations between the amount of regular physical exercise or physical activity and measures of asthma control [6–8]. The World Health Organization defines physical activity as “any bodily movement produced by skeletal muscles that require energy expenditure” and regular exercise as “a subcategory of physical activity that is planned, structured, repetitive, and aims to improve or maintain one or more components of physical fitness” [9]. Heikkinen and colleagues [6] studied the relation between the amount of regular exercise and asthma control among 162 young adults with asthma recruited from the Espoo Cohort Study. Asthma control was assessed by the occurrence of asthma-related symptoms, including wheezing, shortness of breath, cough, and phlegm production during the past 12 months. These were less common among subjects who reported doing regularly moderate exercise. The association was strongest among overweight subjects. Russell and colleagues [7] conducted a longitudinal analysis of the Bergen cohort, assessing the association of baseline physical activity with follow-up asthma, including incident asthma and asthma-related symptoms. Light, but not vigorous, physical activity predicted less follow-up asthma. Loponen and colleagues [8] studied the association between daily physical activity and decline in lung function among 201 patients with adult-onset asthma over a period of 12 years after the asthma diagnosis. They found that the high physical activity group had slower annual FEV1 and FVC decline compared with the low physical activity group. Physical activity was measured at the follow-up only.

The objective of the present study was to elaborate on the relation between the amount of regular exercise and asthma control among adults with diagnosed bronchial asthma representing a wide age range of adult asthma. We also wanted to investigate, whether a similar pattern to that we had detected among young Finnish adults in the Espoo Cohort Study [6] would be observable among adults with a broader age range, as the study population of this Northern Finnish Asthma Study has, and in the colder outdoor climate of Northern Finland.

## Methods

### Study design and study population

The Northern Finnish Asthma Study (ie. NoFAS) is a population-based cross-sectional study of adults 17–73 years old, who had bronchial asthma and who were living in Northern Finland in 2012 [10]. With Northern Finland we mean here the Oulu University Hospital District, i.e. the Northern Ostrobothnia and Lapland.

In 2012, a random sample of 5000 adult subjects, who had the diagnosis of bronchial asthma according to the Finnish National Social Insurance Institution, i.e. KELA (which warrants the special reimbursement right for asthma medication nationally), received two self-administered questionnaires sent by the KELA: the NoFAS questionnaire designed specifically for this study and the St George’s Respiratory Questionnaire. A total of 2033 subjects (41%) responded to the questionnaires. We excluded subjects whose age or gender was unknown, so the final study population was 1995 (40%) adult subjects. The study protocol was approved by the Ethics Committee of the Oulu University Hospital District.

### Determinant of interest

The main determinant of interest was the amount of weekly regular exercise during leisure time. This was asked in the NoFAS Questionnaire with the following question: During the past 12 months, how many hours per week did you exercise on average in your leisure time? (Mark 0 if not at all). 1) Strenuous exercise (e.g. running) ______h/week, 2) Moderate exercise (e.g. jogging or cycling) ______h/week, and 3) Light exercise (e.g. brisk walking or light exercise at gym) _______h/week. All these three forms of exercise were summed and the total amount of hours of exercise per week during leisure time formed the main determinant of interest.

### Outcome

The outcome was asthma control. The study subjects were asked to fill in the Asthma Control Test (ACT) [11]. The NoFAS questionnaire included the five questions used for assessing the ACT score. These were: 1) During the past 4 weeks, how much of the time did your asthma keep you from getting much done at work, at school or at home? (All of the time, most of the time, some of the time, a little of the time, none of the time). 2. During the past 4 weeks, how often did you experience shortness of breath? (More than once a day, once a day, 3 to 6 times a week, once or twice a week, not at all). 3. During the past 4 weeks, how often did your asthma symptoms (wheezing, cough, shortness of breath, chest tightness or pain) wake you up at night or earlier than usual in the morning? (4 or more nights a week, 2 or 3 nights a week, once a week, once or twice, not at all). 4. During the past 4 weeks, how often have you needed your rescue inhaler or nebulizer asthma medication (such as salbutamol)? (3 or more times per day, 1 to 2 times per day, 2 or 3 times per week, once a week or less frequently, not at all). 5. How would you rate your asthma control during the past 4 weeks? (Not at all controlled, poorly controlled, somewhat controlled, well controlled, completely controlled). The ACT score was calculated by summing the points according to the answers to all the five questions described above. For each question, the first choice provided 1 point and the last choice 5 points, so the ACT could have total scores from 5 to 25, where 5 indicated uncontrolled asthma and 25 completely controlled asthma.

### Statistical methods

We estimated the relation between the amount of regular exercise during leisure time, in hours per week, and asthma control, assessed with the ACT score and its five items. In the analyses, we compared the mean ACT score between the five categories of the amount of weekly exercising: 1) no; 2) low (>0 to ≤2h); 3) medium (>2 to ≤ 5h); 4) high (>5 to ≤ 10h); and 5) very high (>10h). Because our previous findings [6] and current preliminary analyses indicated that the relation of interest was non-linear, we estimated the mean ACT score in different categories of exercise applying the analysis of variance ANOVA [12]. We applied Poisson regression analysis using identity link function [12] to estimate the difference in ACT between the optimal exercise category with the best mean ACT and the other exercise categories. In men, this reference category was Medium (>2 to < 5h per week) and in women and in the total population, it was High (>5 to <10h per week). In these analyses, we adjusted for gender, age, education, Body Mass Index (BMI), active smoking, and exposure to second-hand smoke (SHS). We elaborated on the role of the intensity of regular exercise by assessing the effects of the amount of moderate and strenuous exercise. The amount and intensity were strongly correlated, which restricted the study of the independent effects of intensity. We also estimated the adjusted risk ratios (RRs) separately for the five ACT items by contrasting each exercise category to the reference category. For these analyses, we applied Poisson regression with a logarithmic link function. These analyses were carried out using the GENMOD-procedure in the SAS software (SAS 9.3, SAS Institute, North Carolina).

## Results

### Characteristics of the study population

In the present analyses, the total NoFAS study population included 1922 subjects (response rate 38%), because 73 subjects did not provide information on exercise habits. Among this total study population, 65.5% were women and 34.5% were men. Characteristics of the NoFAS population are presented in Table 1 according to the different exercise categories. The amount of weekly exercise was greater among men, young, subjects with normal BMI and high education, never smokers, and those who were less exposed to second hand smoke.

**Table 1:** Characteristics of the study population (n = 1995 *). The Northern Finnish Asthma Study (NoFAS).

	Exercise (hours per week)					
Characteristic	No (0 h)	Low (> 0 ≤ 2 h)	Medium (>2 ≤ 5 h)	High (> 5 ≤ 10 h)	Very high (>10 h)	Total
**Study population, N (%)**	472 (24.56)	266 (13.84)	466 (24.25)	456 (23.73)	262 (13.63)	1922 (100.00)
**Age**						
< 30	11 (2.33)	29 (10.90)	52 (11.16)	82 (17.98)	36 (13.74)	210 (10.93)
30–39	37 (7.84)	50 (18.80)	69 (14.81)	74 (16.23)	34 (12.98)	264 (13.74)
40–49	83 (17.58)	56 (21.05)	90 (19.31)	71 (15.57)	49 (18.70)	349 (18.16)
50–59	168 (35.59)	77 (28.95)	147 (31.55)	140 (30.70)	86 (32.82)	618 (32.15)
> 60	173 (36.65)	54 (20.30)	108 (23.18)	89 (19.52)	57 (21.76)	481 (25.03)
Missing	73					
**Gender**						
Men	146 (30.93)	73 (27.44)	139 (29.83)	183 (40.13)	122 (46.56)	663 (34.50)
Women	326 (69.07)	193 (72.56)	327 (70.17)	273 (59.87)	140 (53.44)	1259 (65.50)
Missing	73					
**Body mass index**						
< 20	12 (2.59)	14 (5.34)	21 (4.58)	22 (4.92)	11 (4.25)	80 (4.23)
20–25	109 (23.54)	66 (25.19)	164 (35.73)	165 (36.91)	107 (41.31)	611 (32.33)
25–30	179 (38.66)	97 (37.02)	161 (35.08)	162 (36.24)	88 (33.98)	687 (36.35)
30–35	104 (22.46)	58 (22.14)	76 (16.56)	77 (17.23)	40 (15.44)	355 (18.78)
> 35	59 (12.74)	27 (10.31)	37 (8.06)	21 (4.70)	13 (5.02)	157 (8.31)
Missing	105					
**Education**						
Comprehensive or upper secondary	157 (33.40)	47 (17.80)	91 (19.53)	101 (22.15)	69 (26.44)	465 (24.26)
Vocational school	240 (51.06)	150 (56.82)	241 (51.72)	222 (48.68)	139 (53.26)	992 (51.75)
Academic degree	73 (15.53)	67 (25.38)	134 (28.76)	133 (29.17)	53 (20.31)	460 (24.00)
Missing	78					
**Smoking**						
Never smoker	198 (42.13)	131 (49.81)	260 (55.91)	253 (55.97)	146 (56.37)	988 (51.75)
Ex-smoker	153 (32.55)	87 (33.08)	142 (30.54)	132 (29.20)	64 (24.71)	578 (30.28)
Current smoker	119 (25.32)	45 (17.11)	63 (13.55)	67 (14.82)	49 (18.92)	343 (17.97)
Missing	86					
**Second hand smoke**						
Yes	288 (61.67)	162 (61.36)	297 (64.01)	307 (67.77)	184 (71.32)	1238 (64.95)
No	179 (38.33)	102 (38.64)	167 (35.99)	146 (32.23)	74 (28.68)	668 (35.05)
Missing	89					

*Information on exercise missing for 2 subjects

### The relation between the amount of exercise and asthma control

Table 2 presents the mean of the unadjusted ACT score and its five items for each exercise category in the total NoFAS study population, as well as separately for men and women. The mean ACT score in the total study population indicated good average asthma control at 20.3 (95% CI 20.2–20.5), similar in women (mean 20.4, 95% CI 20.13–20.59) and men (mean 20.3, 95% CI 20.15–20.52). The ACT score increased gradually, i.e. asthma control improved, by an increase in the amount of weekly exercise from no exercise (mean 19.4, 95% CI 19.95–19.81) to high exercise category (mean 21.0, 95% CI 20.59–21.30), but was slightly lower (mean 20.3, 95% CI 19.79–20.82) in the very high exercise category (>10 h per week). This non-linear relation was present both in men and women, the optimal amount of exercise being slightly different: >2 ≤5 h among men (with ACT 20.86, 95% CI 20.27–21.44) and >5 ≤10 h among women (mean 21.97, 95% CI 21.11 to 22.82).

**Table 2:** The Asthma Control Test score according to the amount of regular exercise (in h/week).

		Exercise (hours per week)					
Symptom	No (0 h)	Low (> 0 ≤ 2 h)	Medium (>2 ≤ 5 h)	High (> 5 ≤ 10 h)	Very high (>10 h)	Missing	Total
**Study population, N (%)**	472 (24.56)	266 (13.84)	466 (24.25)	456 (23.73)	262 (13.63)		1922 (100.0)
***Asthma Control Test***	**Mean** **(95% CI)**	**Mean** **(95% CI)**	**Mean** **(95% CI)**	**Mean** **(95% CI)**	**Mean** **(95% CI)**	**Mean** **(95% CI)**	**Mean** **(95% CI)**
ACT Score	19.38 (18.95–19.81)	20.50 (19.98–21.02)	20.81 (20.47–21.15)	20.95 (20.59–21.30)	20.30 (19.79–20.82)	18.70 (17.37–20.03)	20.33 (20.15–20.52)
Getting things done at work, school or both ^1^	3.99 (3.88–4.10)	4.26 (4.13–4.38)	4.37 (4.29–4.46)	4.40 (4.32–4.49)	4.26 (4.14–4.39)	4.00 (3.72–4.28)	4.25 (4.20–4.29)
Having shortness of breath ^2^	3.89 (3.78–4.00)	4.08 (3.95–4.22)	4.11 (4.02–4.21)	4.20 (4.11–4.30)	4.10 (3.96–4.23)	3.65 (3.29–4.00)	4.06 (4.01–4.11)
Wake up at night due to asthma symptoms ^3^	4.13 (4.01–4.25)	4.37 (4.23–4.51)	4.42 (4.32–4.51)	4.36 (4.25–4.46)	4.24 (4.09–4.38)	3.89 (3.55–4.23)	4.29 (4.24–4.34)
Use of rescue inhaler or nebulizer medication ^4^	3.51 (3.39–3.63)	3.76 (3.61–3.91)	3.84 (3.74–3.95)	3.84 (3.74–3.95)	3.67 (3.53–3.81)	3.33 (3.02–3.65)	3.71 (3.66–3.77)
Rating of asthma control^5^	3.80 (3.71–3.88)	4.00 (3.89–4.11)	4.02 (3.94–4.10)	4.10 (4.03–4.18)	4.03 (3.93–4.14)	3.64 (3.43–3.86)	3.97 (3.93–4.01)
**Men**							
***Asthma Control Test***	**Mean** **(95% CI)**	**Mean** **(95% CI)**	**Mean** **(95% CI)**	**Mean** **(95% CI)**	**Mean** **(95% CI)**	**Mean** **(95% CI)**	**Mean** **(95% CI)**
ACT Score	19.30 (18.49–20.11)	19.91 (18.86–20.97)	21.02 (20.41–21.63)	20.86 (20.27–21.44)	20.42 (19.67–21.17)	17.68 (15.68–19.69)	20.28 (19.96–20.61)
Getting things done at work, school or both ^1^	3.88 (3.66–4.10)	4.08 (3.82–4.35)	4.39 (4.24–4.55)	4.36 (4.21–4.50)	4.31 (4.14–4.49)	3.79 (3.33–4.26)	4.21 (4.12–4.29)
Having shortness of breath ^2^	3.83 (3.60–4.05)	4.07 (3.81–4.33)	4.21 (4.04–4.38)	4.20 (4.05–4.35)	4.10 (3.91–4.29)	3.42 (2.81–4.03)	4.062 (3.98–4.15)
Wake up at night due to asthma symptoms ^3^	4.20 (4.00–4.40)	4.24 (3.94–4.54)	4.57 (4.41–4.73)	4.41 (4.26–4.56)	4.23 (4.01–4.45)	3.46 (2.84–4.08)	4.31 (4.22–4.40)
Use of rescue inhaler or nebulizer medication ^4^	3.57 (3.36–3.78)	3.56 (3.28–3.85)	3.76 (3.55–3.97)	3.77 (3.60–3.95)	3.67 (3.48–3.91)	3.07 (2.56–3.59)	3.66 (3.57–3.76)
Rating of asthma control^5^	3.75 (3.59–3.91)	3.93 (3.72–4.13)	4.09 (3.95–4.23)	4.07 (3.95–4.19)	4.11 (3.95–4.27)	3.44 (3.13–3.76)	3.97 (3.91–4.04)
**Women**							
***Asthma Control Test***	**Mean** **(95% CI)**	**Mean** **(95% CI)**	**Mean** **(95% CI)**	**Mean** **(95% CI)**	**Mean** **(95% CI)**	**Mean** **(95% CI)**	**Mean** **(95% CI)**
ACT Score	19.41 (18.91–19.92)	20.72 (20.13–21.32)	20.72 (20.30–21.14)	21.01 (20.55–21.46)	20.20 (19.48–20.92)	19.34 (17.54–21.15)	20.36 (20.13–20.59)
Getting things done at work school or both ^1^	4.04 (3.91–4.16)	4.32 (4.18–4.46)	4.36 (4.26–4.46)	4.44 (4.34–4.54)	4.22 (4.04–4.40)	4.13 (3.76–4.49)	4.27 (4.21–4.32)
Having shortness of breath ^2^	3.92 (3.79–4.05)	4.09 (3.93–4.25)	4.07 (3.96–4.18)	4.21 (4.09–4.32)	4.09 (3.91–4.28)	3.78 (3.34–4.22)	4.06 (4.00–4.12)
Wake up at night due to asthma symptoms ^3^	4.10 (3.96–4.24)	4.42 (4.26–4.57)	4.35 (4.24–4.47)	4.32 (4.19–4.46)	4.24 (4.05–4.43)	4.18 (3.79–4.57)	4.27 (4.21–4.34)
Use of rescue inhaler or nebulizer medication ^4^	3.48 (3.34–3.63)	3.84 (3.67–4.01)	3.88 (3.76–4.00)	3.89 (3.75–4.02)	3.65 (3.45–3.84)	3.50 (3.09–3.91)	3.74 (3.67–3.80)
Rating of asthma control^5^	3.82 (3.72–3.91)	4.03 (3.90–4.15)	3.99 (3.90–4.08)	4.12 (4.03–4.22)	3.97 (3.83–4.12)	3.78 (3.48–4.07)	3.97 (3.92–4.02)

^1^ In the past 4 weeks, how much of the time did your asthma keep you from getting much done at work, school or at home? All of the time (1 point); Most of the time (2); Some of the time (3); A little of the time (4); None of the time (5 points).

^2^ During the past 4 weeks, how often have you had shortness of breath? More than once a day (1); Once a day (2); 3 to 6 times a week (3); Once or twice a week (4); Not at all (5).

^3^During the past 4 weeks, how often did your asthma symptoms (wheezing, coughing, shortness of breath, chest tightness or pain) wake you up at night or earlier than usual in the morning? 4 or more nights a week (1); 2 to 3 nights a week (2); Once a week (3); Once or twice (4); Not at all (5).

^4^ During the past 4 weeks, how often have you used your rescue inhaler or nebulizer medication (such as salbutamol)? 3 or more times per day (1); 1 or 2 times per day (2); 2 or 3 times per week (3);/ Once a week or less (4); Not at all (5).

^5^ How would you rate your asthma control during the past 4 weeks? Not controlled at all (1); Poorly controlled (2); Somewhat controlled (3); Well controlled (4); Completely controlled (5).

Table 2 also shows the results for all individual items forming the ACT score, the results are presented for each exercise category. The scale for each variable was from 1 (i.e. poor asthma control) to 5 (i.e. completely controlled asthma). In the total population, the highest score (i.e. the best situation of asthma control) is seen in the High exercise category for all individual items.

When looking at these scores separately among men and women, the following trends were detected: Among men, the highest ACT score (i.e. best asthma control) was detected in the Medium exercise category for all the individual items, with a decreasing trend from Medium to the lower exercise categories, as well as slightly decreasing scores from Medium to High and Very High exercise categories. Among women, the best score was detected in the High exercise category for all individual items, with a slightly smaller score in the Very high exercise category, as well as with decreasing scores when going towards smaller exercise.

Table 3 presents the effect estimates for the effect of regular exercise on asthma control from Poisson regression analyses, adjusting for age, education, Body Mass Index, personal smoking, and exposure to second hand smoke. Using the optimal exercise category as the reference (high in all combined and women, and medium in men), the effect estimate presents the difference in ACT score between the reference and the exercise category of interest. In the total population, ACT score decreased 1.08 points (95% CI 0.50–1.66) in the No exercise category and it decreased 0.53 points (95% CI -0.09–1.14) in the Very high category compared to the High exercise category forming the reference. Among men, the reference category with the best ACT score was Medium weekly exercise, and the ACT score decreased with both less weekly exercise as well as with more weekly exercise. In women, both the effect estimate for No exercise (difference -1.17, 95% CI -1.86 to– 0.46) and Very high exercise (difference -0.89, 95% CI -1.74 to -0.04) were statistically significantly lower compared to the reference category of High weekly exercise.

**Table 3:** The effect of regular exercise on the Asthma Control Test score. The estimates are from Generalized Linear Models with Poisson distribution and identity link and present deviation in ACT points from the optimal reference exercise category with the best ACT score.

	Exercise (hours per week)				
Model	No (0 h)	Low (> 0 ≤ 2 h)	Medium (>2 ≤ 5 h)	High (> 5 ≤ 10 h)	Very high (>10 h)
**Total population**	Difference 95% CI	Difference 95% CI	Difference 95% CI	Reference	Difference 95% CI
**Crude**	-1.57 -2.12 to -1.01	-0.44 -1.07 to 0.19	-0.13 -0.63 to 0.36	20.95 20.59 to 21.30	-0.64 -1.27 to -0.02
Adjusted*	-1.08 -1.66 to -0.50	-0.10 -0.72 to 0.52	-0.04 -0.53 to 0.45	22.08 21.40 to 22.77	-0.53 -1.14 to 0.09
**Men**	Difference 95% CI	Difference 95% CI	Reference	Difference 95% CI	Difference 95% CI
**Crude**	-1.72 -2.72 to -0.71	-1.10 -2.30 to 0.09	21.01 20.41 to 21.62	-0.16 -1.00 to 0.67	-0.60 -1.55 to 0.36
Adjusted*	-1.28 -2.30 to -0.25	-0.53 -1.71 to 0.65	22.67 21.40 to 23.94	-0.34 -1.15 to 0.46	-0.31 -1.24 to 0.62
**Women**	Difference 95% CI	Difference 95% CI	Difference 95% CI	Reference	Difference 95% CI
**Crude**	-1.59 -2.27 to -0.91	-0.28 -1.02 to 0.46	-0.28 -0.90 to 0.33	21.01 20.55 to 21.46	-0.81 -1.65 to 0.03
Adjusted*	-1.17 -1.89 to -0.46	-0.09 -0.84 to 0.66	-0.21 -0.84 to 0.41	21.97 21.11 to 22.82	-0.89 -1.74 to -0.04

* Adjusted for age, Body Mass Index, personal smoking, exposure to second hand smoke and education.

A total of 86.99% of the subjects reported some moderate exercise, whereas 46.05% experienced some strenuous exercise. There was a strong correlation between the intensity of exercise and the amount of regular exercise in hours per week. Participants in the highest exercise category (> 10 h per week) reported a mean of 7.79 (SD 7.81) hours per week of strenuous exercise, whereas in the low exposure category (> 0 to 2 hours) the corresponding mean was 0.34 (SD 0.59) hours per week.

Table 4 presents both the crude and adjusted effect estimates of regular exercise on the five items of the Asthma Control Test score from Poisson regression models. The effect estimates for the contrast between No exercise and the reference are consistently negative and statistically significant. The effect estimates for the contrast between Very high and the reference categories are also consistently negative, but weaker and not always statistically significant due smaller group size.

**Table 4:** The effect of regular exercise on the five items of the Asthma Control Test score. The estimates are from Generalized Linear Models with Poisson distribution and identity link function and present deviation in ACT points from the optimal reference exercise category showing the best ACT score. Adjusted for age, Body Mass Index, personal smoking, exposure to second hand smoking and education.

	Exercise (hours per week)				
Symptom	No (0 h)	Low (> 0–2 h)	Medium (>2 ≤ 5 h)	High (> 5 ≤ 10 h)	Very high (>10 h)
Study population, N (%)	472 (24.56)	266 (13.84)	466 (24.25)	456 (23.73)	262 (13.63)
***Asthma Control Test***				Reference	
Getting things done at work, school or home ^1^, crude	-0.41 -0.55 to -0.28	-0.15 -0.30 to 0.00	-0.03 -0.15 to 0.08	4.40 4.32 to 4.49	-0.14 -0.29 to 0.01
Getting things done at work, school or home ^1^, adjusted	-0.33 -0.48 to -0.19	-0.08 -0.23 to 0.07	-0.03 -0.15 to 0.09	4.73 4.56 to 4.89	-0.12 -0.27 to 0.03
Having shortness of breath ^2^, crude	-0.31 -0.46 to -0.17	-0.12 -0.28 to 0.04	-0.09 -0.22 to 0.04	4.20 4.11 to 4.30	-0.11 -0.27 to 0.05
Having shortness of breath ^2^, adjusted	-0.23 -0.38 to -0.08	-0.05 -0.21 to 0.11	-0.08 -0.21 to 0.05	4.53 4.34 to 4.72	-0.10 -0.26 to 0.07
Wake up at night due to asthma symptoms ^3^, crude	-0.23 -0.38 to -0.07	0.01 -0.16 to 0.18	0.06 -0.08 to 0.20	4.36 4.25 to 4.46	-0.12 -0.30 to 0.05
Wake up at night due to asthma symptoms ^3^, adjusted	-0.11 -0.27 to 0.05	0.10 -0.07 to 0.27	0.10 -0.04 to 0.24	4.50 4.31 to 4.69	-0.08 -0.25 to 0.10
Use of rescue inhaler or nebulizer medication ^4^, crude	-0.33 -0.49 to -0.17	-0.08 -0.26 to 0.10	0.00 -0.15 to 0.15	3.84 3.74 to 3.95	-0.17 -0.35 to 0.01
Use of rescue inhaler or nebulizer medication ^4^, adjusted	-0.20 -0.37 to -0.04	-0.03 -0.21 to 0.16	0.01 -0.13 to 0.16	3.98 3.78 to 4.19	-0.15 -0.33 to 0.03
Rating of asthma control^5^, crude	-0.31 -0.42 to -0.19	-0.10 -0.23 to 0.03	-0.08 -0.19 to 0.02	4.10 4.02 to 4.18	-0.07 -0.20 to 0.06
Rating of asthma control^5^, adjusted	-0.25 -0.37 to -0.13	-0.07 -0.20 to 0.07	-0.09 -0.19 to 0.02	4.29 4.15 to 4.44	-0.06 -0.19 to 0.07
**Men**					
***Asthma Control Test***			Reference		
Getting things done at work, school or home ^1^, crude	-0.51 -0.78 to -0.24	-0.31 -0.61 to -0.01	4.39 4.24 to 4.54	-0.04 -0.25 to 0.17	-0.08 -0.31 to 0.15
Getting things done at work, school or home ^1^, adjusted	-0.44 -0.72 to -0.16	-0.17 -0.47 to 0.13	4.79 4.47 to 5.10	-0.06 -0.26 to 0.15	-0.01 -0.25 to 0.22
Having shortness of breath ^2^, crude	-0.39 -0.66 to -0.11	-0.14 -0.45 to 0.16	4.21 4.04 to 4.38	-0.01 -0.24 to 0.21	-0.11 -0.37 to 0.14
Having shortness of breath ^2^, adjusted	-0.29 -0.58 to 0.00	0.00 -0.31 to 0.31	4.79 4.43 to 5.15	-0.05 -0.28 to 0.18	-0.07 -0.33 to 0.18
Wake up at night due to asthma symptoms ^3^, crude	-0.37 -0.62 to -0.11	-0.33 -0.66 to 0.00	4.57 4.41 to 4.73	-0.16 -0.38 to 0.05	-0.34 -0.61 to -0.07
Wake up at night due to asthma symptoms ^3^, adjusted	-0.32 -0.59 to -0.06	-0.23 -0.55 to 0.09	4.77 4.42 to 5.12	-0.20 -0.42 to 0.01	-0.28 -0.54 to -0.02
Use of rescue inhaler or nebulizer medication ^4^, crude	-0.19 -0.49 to 0.10	-0.20 -0.54 to 0.15	3.76 3.56 to 3.97	0.01 -0.25 to 0.28	-0.06 -0.36 to 0.23
Use of rescue inhaler or nebulizer medication ^4^, adjusted	-0.08 -0.37 to 0.21	-0.08 -0.44 to 0.28	4.00 3.62 to 4.38	0.01 -0.26 to 0.27	0.02 -0.29 to 0.32
Rating of asthma control^5^, crude	-0.33 -0.54 to -0.12	-0.16 -0.40 to 0.09	4.09 3.95 to 4.23	-0.02 -0.20 to 0.17	0.02 -0.19 to 0.23
Rating of asthma control^5^, adjusted	-0.26 -0.47 to -0.05	-0.07 -0.31 to 0.18	4.34 4.08 to 4.61	-0.04 -0.22 to 0.14	0.07 -0.14 to 0.28
**Women**					
***Asthma Control Test***				Reference	
Getting things done at work, school or home ^1^, crude	-0.40 -0.56 to -0.24	-0.12 -0.29 to 0.06	-0.08 -0.22 to 0.07	4.44 4.34 to 4.54	-0.22 -0.42 to -0.01
Getting things done at work, school or home ^1^, adjusted	-0.32 -0.49 to -0.15	-0.08 -0.25 to 0.10	-0.07 -0.22 to 0.07	4.73 4.53 to 4.94	-0.24 -0.45 to -0.03
Having shortness of breath ^2^, crude	-0.29 -0.46 to -0.12	-0.12 -0.32 to 0.08	-0.14 -0.30 to 0.02	4.21 4.09 to 4.32	-0.11 -0.33 to 0.10
Having shortness of breath ^2^, adjusted	-0.22 -0.41 to -0.04	-0.08 -0.27 to 0.12	-0.13 -0.30 to 0.03	4.43 4.19 to 4.67	-0.13 -0.35 to 0.09
Wake up at night due to asthma symptoms ^3^, crude	-0.22 -0.42 to -0.03	0.09 -0.11 to 0.30	0.03 -0.15 to 0.21	4.32 4.19 to 4.46	-0.08 -0.31 to 0.15
Wake up at night due to asthma symptoms ^3^, adjusted	-0.09 -0.30 to 0.12	0.16 -0.06 to 0.37	0.06 -0.12 to 0.25	4.46 4.21 to 4.71	-0.08 -0.32 to 0.16
Use of rescue inhaler or nebulizer medication ^4^, crude	-0.40 -0.59 to -0.21	-0.05 -0.26 to 0.17	-0.01 -0.19 to 0.17	3.89 3.75 to 4.02	-0.24 -0.47 to -0.01
Use of rescue inhaler or nebulizer medication ^4^, adjusted	-0.27 -0.48 to -0.07	-0.02 -0.24 to 0.20	0.02 -0.16 to 0.20	3.95 3.70 to 4.20	-0.25 -0.49 to -0.02
Rating of asthma control^5^, crude	-0.31 -0.45 to -0.17	-0.10 -0.26 to 0.06	-0.13 -0.27 to 0.00	4.12 4.03 to 4.22	-0.15 -0.33 to 0.02
Rating of asthma control^5^, adjusted	-0.28 -0.42 to -0.13	-0.09 -0.26 to 0.07	-0.15 -0.28 to -0.01	4.30 4.12 to 4.48	-0.19 -0.37 to -0.02

^1^ In the past 4 weeks, how much of the time did your asthma keep you from getting as much done at work, school or at home? All of the time (1 point); Most of the time (2); Some of the time (3); A little of the time (4); None of the time (5 points).

^2^ During the past 4 weeks, how often have you had shortness of breath? More than once a day (1); Once a day (2); 3 to 6 times a week (3); Once or twice a week (4); Not at all (5).

^3^During the past 4 weeks, how often did your asthma symptoms (wheezing, coughing, shortness of breath, chest tightness or pain) wake you up at night or earlier than usual in the morning? 4 or more nights a week (1); 2 to 3 nights a week (2); Once a week (3); Once or twice (4); Not at all (5).

^4^ During the past 4 weeks, how often have you used your rescue inhaler or nebulizer medication (such as salbutamol)? 3 or more times per day (1); 1 or 2 times per day (2); 2 or 3 times per week (3); Once a week or less (4); Not at all (5).

^5^ How would you rate your asthma control during the past 4 weeks? Not controlled at all (1); Poorly controlled (2); Somewhat controlled (3); Well controlled (4); Completely controlled (5).

## Discussion

In this large population-based study of 1922 adult subjects having bronchial asthma according to the National Social Insurance Institution of Finland (which provides special, higher reimbursement for asthma medications nationally) and living in Northern Finland, we found that in general there is a beneficial effect of regular exercise during leisure time on asthma control. A trend of improving asthma control was seen in the total population from No exercise to High exercise (i.e. >5 to ≤10h per week), while a slight decrease in ACT score was seen in the Very high exercise category (i.e. >10h per week).

Our results suggest that advice about the beneficial effects of regular exercise should be included as an integral part of asthma treatment for adult asthmatic subjects, potentially with the exception of those with severe asthma, as the ACT score decreased slightly in this group among both women and men. Subjects with severe asthma should get special focus in future studies on exercise and asthma control to elaborate what type of exercise is clearly harmful for asthma control in this group and what type of exercise could still be beneficial. Our study population included subjects 17–73 years old, so it has a wide coverage, but potential effects of exercise on asthma control among older asthmatics should be examined in future studies.

Our results question the previous beliefs that exercise would cause exercise-induced asthmatic reactions in the majority of asthmatics. Our results suggest that, on the contrary, regular exercise could and should be included as an integral part of management for most asthmatics, but adequate advice about appropriate type of exercise and use of asthma medications should be provided when exercise is prescribed as part of asthma treatment. In addition, regular follow-up by nurses and/or physicians is recommendable when exercise is prescribed as part of asthma management.

When analyzing the individual components of asthma control test in the total study population, the best asthma control (i.e. the highest score) was detected among the High exercise category for all individual items forming the ACT score. Adjustment for age, gender, smoking, exposure to SHS and education did not influence this trend, although the best adjusted ACT scores were somewhat higher, i.e. better, compared to the unadjusted analyses.

### Validity of results

The strengths of our study include that it was a large population-based study. Adults with asthma were randomly selected from the study area by the National Social Insurance Institution of Finland from its records. KELA provides special (i.e. higher) reimbursement for asthma medication (in comparison to the regular level of reimbursement of medications), when the diagnosis of asthma follows the national guidelines, which provides an economic incentive for people to have their asthma diagnosed in a proper way, i.e. based on the national guidelines in Finland [13].

The response rate was 40%, which is reasonable, but less than usual in our epidemiologic studies [3, 14, 15]. The Finnish law states that when the study subjects are identified through the KELA records, KELA must send the invitation letter along with the study questionnaires, in order to guarantee anonymity. Thus, we were not allowed to send any reminder letters by ourselves. In any case, the relatively large study population of 1995 subjects provides a good picture of the situation in Northern Finland. However, among those not replying to the questions concerning regular exercise, the ACT score was somewhat lower (18.70, 95% CI 17.37–20.03), suggesting that at least some of those with a poorer asthma control did not want to provide information of their exercise habits. Thus, our study may underestimate to some extent the beneficial effects of regular exercise on asthma control.

Our outcome, i.e. asthma control, was assessed based on the self-administered Asthma Control Test (ACT) that has been validated previously [11]. The NoFAS questionnaire included the five questions based on which the ACT score is assessed. Our main determinant of interest was exercise during leisure time that was inquired by three questions, the answers to which were then summed up. Our interest focused on exercise during leisure time, as the amount and type of this form of exercise can be influenced by giving advice by the medical team as part of asthma management. We used the average amount of exercise in hours per week as the main determinant of interest. In addition, we asked the participants to provide information on the intensity of regular exercise. The average amount of exercise in hours per week is an objective measure, whereas the judgement of the intensity is more subjective and possibly related to the physical condition and level of asthma control of the participant. To minimize potential recall bias, we applied the amount of regular exercise as the main determinant of interest.

To control for potential confounding, we adjusted the analyses on the relation between exercise and ACT for age, gender, education, BMI, smoking and exposure to second-hand smoke. Education was considered to be also a proxy for occupation, so it would adjust for potential occupational exposures that would influence asthma control.

### Synthesis with previous knowledge

To our knowledge, this study is the first population-based epidemiological study that addresses potential effects of regular exercise on asthma control among adult asthmatics living in a subarctic or arctic climate. The present study in Northern Finland examined potential effects of regular exercise in a relatively cold climate, especially during wintertime. Findings of all the three epidemiological studies that have been reported previously [6–8] are consistent with our results, i.e. they show a beneficial effect of physical activity or regular exercise on asthma.

Some studies have suggested mechanisms that may underline the beneficial effects of exercise on asthma control and provide mechanistic credibility for our results. Lucas et al. [16] suggested that regular exercise has beneficial effects on the airway smooth muscle layer and on breathing pattern, and that these effects could eventually reduce bronchial hyperresponsiveness. Better physical fitness may also lead to a higher threshold for experiencing breathlessness. Mendes et al. [17] studied 68 asthmatics 20 to 50 years of age in a laboratory setting and showed that aerobic training leads to a significant decrease in airway inflammation, measured by sputum eosinophils and exhaled nitrogen oxide.

### Causal inference

We addressed a causal hypothesis with the assumption that moderate regular exercise would improve control of asthma, but intensive exercise could worsen some of the measures of asthma control [6]. The observed empirical associations that we found between the amount of weekly regular exercise and ACT are consistent with our causal hypothesis. Any major confounding was not likely, because many of the main determinants of asthma control were adjusted for in the analyses. An alternative explanation for the observed associations is that subjects with poor asthma control are inclined to avoid regular exercise. This problem of temporality can not be excluded in the present cross-sectional design. A randomized controlled trial (RCT) can avoid this type of limitation, but may have other problems in causal inference, because the exercise intervention can not be blinded from the participants. The results from available RCTs provide evidence that guided exercise is likely to improve asthma control in a selected group of asthmatics, and thus, complement our study [18].

### Conclusions

In this large population-based study conducted in Northern Finland, we provide evidence that regular exercise improves asthma control in adults, showing a trend of improving asthma control from no exercise to high exercise in the total population. Thus, exercise seems to be beneficial for asthma control even in the relatively cool subarctic and arctic climate such as that in Northern Finland. Our results propose that advising to take up (or keep) regular light to medium or sometimes even heavy exercise among adult subjects with asthma will be useful for them, when targeting at achieving good asthma control.

## Abbreviations

ACT: Asthma Control Test
CI: confidence interval
KELA: Finnish National Social Insurance Institution
NoFAS: Northern Finnish Asthma Study
OR: odds ratio
RR: risk ratio
